# Outcomes of high-risk adult outpatients treated with early remdesivir therapy during the severe acute respiratory syndrome coronavirus 2 (SARS-CoV-2) omicron era: experiences from the national centre of Hungary

**DOI:** 10.1007/s00210-023-02456-y

**Published:** 2023-03-09

**Authors:** Zsófia Gáspár, Bálint Gergely Szabó, Anita Ábrahám, Zsuzsanna Várnai, Noémi Kiss-Dala, János Szlávik, János Sinkó, István Vályi-Nagy, Botond Lakatos

**Affiliations:** 1 School of PhD Studies, Semmelweis University, Üllői Street 26., 1085 Budapest, Hungary; 2 Departmental Group of Infectious Diseases, Department of Internal Medicine and Haematology, Semmelweis University, Albert Flórián Street 5-7., 1097 Budapest, Hungary; 3 National Institute of Haematology and Infectious Diseases, South Pest Central Hospital, Albert Flórián Street 5-7., 1097 Budapest, Hungary

**Keywords:** COVID-19, Coronavirus disease 2019, SARS-CoV-2, Severe acute respiratory syndrome coronavirus 2, Omicron, Remdesivir, Outpatient, Drug

## Abstract

Emerging evidence suggests that remdesivir might improve clinical outcome of high-risk outpatients with coronavirus disease 2019 (COVID-19). Our aim was to evaluate characteristics and outcomes of nonhospitalised adults diagnosed with COVID-19 and treated with early remdesivir therapy during the omicron wave. A single-centre prospective cohort study was performed among adult patients between February and June 2022, during the circulation of phylogenetic assignment of named global outbreak (PANGO) subvariants BA.2, BA.4, and BA.5 in Hungary. Patients were enrolled based on pre-defined criteria. Clinical characteristics (demography, comorbidities, vaccination status, imaging, treatment, and disease course) and outcomes (COVID-19 related hospitalisation, oxygen supplementation, intensive care support, and all-cause death) were assessed at 28 days post-treatment. A subgroup analysis of patients with and without active haematological malignancies was also carried out. Altogether, 127 patients were enrolled: 51.2% (65/127) were female with a median age of 59 (IQR: 22, range: 21‒92) years, and 48.8% (62/127) had active haematological malignancy. At 28 days post-treatment, 7.1% (9/127) of patients required COVID-19-related hospitalisation, 2.4% (3/127) required oxygen supplementation, 1.6% (2/127) required intensive care, and 0.8% (1/127) died due to a non-COVID-19-related secondary infection at the intensive care unit, all with haematological malignancies. Early remdesivir treatment might be a feasible strategy among high-risk outpatients with COVID-19 during the omicron wave.

**Supplementary Information:**

The online version contains supplementary material available at 10.1007/s00210-023-02456-y.

## Introduction

Management strategies of patients infected with severe acute respiratory syndrome coronavirus 2 (SARS-CoV-2) depend on the clinical context, patient-level risk factors, and treatment availability (IDSA 2022). Outpatients who are unvaccinated or partially immunised, as well as elderly, obese, or immunocompromised are at higher risk for severe or progressive disease (Gottlieb et al. 2022). Therefore, early initiation of therapy might be warranted in these subpopulations (Gottlieb et al. 2022).

Antiviral treatment options include monoclonal antibodies and direct antiviral agents (IDSA 2022). Monoclonal antibodies targeting spike protein of the SARS-CoV-2 have been shown to provide clinical benefit among certain clinical circumstances in treating coronavirus disease 2019 (COVID-19). However, the anticipated in vivo activity of different monoclonal antibodies greatly varies depending on the viral subvariant type, therefore limiting universal applicability (IDSA 2022). Currently, recommended antiviral drugs against SARS-CoV-2 are polymerase inhibitors remdesivir, molnupiravir, and the protease inhibitor combination nirmatrelvir/ritonavir (IDSA 2022). Remdesivir has already been licenced for in-hospital treatment of SARS-CoV-2-infected patients, but recent data support its use in early COVID-19 in the outpatient setting by lowering the overall risk of hospitalisation and disease progression (Lin et al. 2021; Panagopoulos et al. 2022).

Access to the different monoclonal antibodies and direct antiviral agents varies by country. In Hungary, particularly in the context of lacking oral antivirals during our study period, remdesivir became the single therapeutic option for high-risk outpatients. The aim of the present study was to evaluate clinical characteristics and outcomes of high-risk adults receiving early remdesivir treatment in the outpatient setting during the phylogenetic assignment of named global outbreak (PANGO) BA.2, BA.4, and BA.5 omicron subvariant predominance in Hungary (ECDC 2022).

## Materials and methods

### Study design and setting

A single-centre prospective cohort study was conducted among SARS-CoV-2-infected patients who were at high risk for disease progression and received early remdesivir therapy between February and June 2022. Our centre is a national-level referral institution of COVID-19 with a high-influx COVID-19 outpatient department during the pandemic (Supplementary File 1). The study design was in accordance with the Helsinki Declaration and national ethical standards. The study protocol was approved by the Institutional Review Board of South Pest Central Hospital, National Institute of Haematology and Infectious Diseases (IKEB-14/2020). Written informed consent was obtained from each included patient.

### Patient eligibility and inclusion

Eligible high-risk patients were aged at least 18 years, had ongoing COVID-19 confirmed by SARS-CoV-2 nasopharyngeal sample positivity using real-time polymerase chain reaction (RT-PCR) and antigenic testing, and possessed ≥ 1 predefined patient-level risk factor for disease progression at inclusion. Predefined patient-level risk factors were essential hypertension, obesity (body mass index > 25 kg/m^2^), chronic cardiovascular disease, chronic cerebrovascular disease, chronic pulmonary disease, chronic renal disease, chronic liver disease, diabetes mellitus, immunocompromised state, and active oncological and haematological malignancies. All eligible patients were included consecutively at COVID-19 diagnosis if they consented to receive remdesivir for a minimum of 3 days and promptly started after diagnosis ascertainment. No a priori exclusion criteria were used. COVID-19 severity was given according to the World Health Organisation (WHO) criteria (WHO 2022). After inclusion completion, the cohort was stratified into two subgroups according to active haematological malignancy as a comorbidity.

### Data collection

Patient data were collected anonymously through electronic medical records and clinical charts and recorded in a structural database. Data collected were 1) age and sex, 2) comorbidities, 3) COVID-19 vaccination status and previous SARS-CoV-2 infections, 4) disease course (onset of typical symptoms, disease severity), 5) nasopharyngeal and blood SARS-CoV-2 RT-PCR results, 6) results of chest computed tomography (CT) scans, 7) details of remdesivir treatment, and 8) clinical outcomes. Baseline variables were recorded at COVID-19 diagnosis.

### Therapeutic strategies

Included patients received 200 mg remdesivir *quaque die* (QD) intravenously and diluted in 0.9% saline according to instructions of the manufacturer, and 100 mg remdesivir QD on the following days. The protocol suggested 3 days of standard treatment, starting on the day of COVID-19 diagnosis, but at the discretion of the attending physician, prolongation of treatment was permitted for a total of 5 days.

### Outcomes and patient follow-up

The primary endpoint was the need for hospitalisation due to COVID-19. Secondary endpoints were all-cause death, need for oxygen supplementation, and need for intensive care unit admission. All outcome measures were assessed at + 28 days after the end of remdesivir treatment. Patient follow-up is detailed in Supplementary File 1*.*

### Statistical analysis

Continuous variables are reported by median with interquartile range (IQR) and minimum–maximum ranges. The Shapiro–Wilk test was used for normality check. Categorical variables are reported as absolute numbers (*n*) with relative percentages (%). Student's *t*-test and chi-squared test were used for statistical comparison depending on variable distribution. Statistical significance was decided at a two-sided *p*-value of < 0.05 for all tests.

## Results

In total, 127 patients were enrolled during the study period (Table 1). Among them, 48.8% (*n* = 62) had an active haematological malignancy. These were non-Hodgkin lymphoma (*n* = 25; 40.3%), acute leukaemia (*n* = 16; 25.8%), chronic lymphocytic leukaemia (*n* = 10; 16.1%), myeloproliferative neoplasms (*n* = 4; 6.5%), Hodgkin’s lymphoma (*n* = 4; 6.5%), and myelodysplastic syndrome (*n* = 3; 4.8%); 51.2% (*n* = 65) of patients had a patient-level risk factor other than haematological malignancy. Median age at diagnosis was 59 (IQR: 22, range: 21–92) years, 51.2% (*n* = 65) of included patients were female in the total cohort. Age, sex, and comorbidities were equally distributed between subgroups, except for essential hypertension, and active oncological and systematic autoimmune diseases, which were statistically more frequent in the non-haematological subgroup. Presence of SARS-CoV-2 RNAemia was found at similar rates between subgroups. Upon chest CT examinations, eighteen patients (17.6%) in the total cohort had alterations typical for COVID-19. More patients were fully vaccinated in the non-haematological subgroup. The severity of symptoms was balanced. The median time from symptom onset to first dose of remdesivir was 3 (IQR: 3, range: 0–63) days, with no statistically significant difference between subgroups. From the total cohort, nine patients (7.1%) had to be admitted to the hospital, three (2.4%) required oxygen supplementation, two (1.6%) were transferred to intensive care unit, and one (0.8%) died within the study period. All negative outcomes occurred among patients with haematological malignancy. The 3-day therapy protocol was more frequently administered among non-haematological patients (38.6% vs. 12.9%, *p* < 0.01).

**Table 1:** Baseline characteristics and outcomes of patients included in the study

Parameter	Total (*n* = 127)	Haematological patients (*n* = 62)	Non-haematological patients (*n* = 65)	*p-*value
Age (years, median; IQR; min–max)	59; 22; 21–92	57; 24; 21–82	63; 21; 31–92	0.24
Male (*n*, %)	62; 48.8%	32; 51.6%	30; 46.2%	0.54
Comorbidities (*n*, %):				
Essential hypertension	55; 43.3%	15; 24.2%	40; 61.5%	< 0.01
Chronic cardiovascular disease	9; 7.1%	2; 3.2%	7; 10.8%	0.10
Chronic pulmonary disease	12; 9.4%	3; 4.8%	9; 13.8%	0.08
Chronic renal disease	3; 2.4%	1; 1.6%	2; 3.1%	0.59
Chronic liver disease	3; 2.4%	0; 0%	3; 4.6%	0.09
Chronic cerebrovascular disease	1; 0.79%	0; 0%	1; 1.5%	0.33
Diabetes mellitus	25; 19.7%	9; 14.5%	16; 24.6%	0.15
Active oncological malignancy	20; 15.7%	3; 4.8%	17; 26.1%	< 0.01
Active haematological malignancy	62; 48.8%	62; 100%	0; 0%	n.a
Systematic autoimmune disease	17; 13.4%	4; 6.5%	13; 20.0%	< 0.01
Chronic systemic corticosteroid treatment	4; 3.2%	2; 3.2%	2; 3.1%	0.96
Chronic immunosuppressive drug use	14; 11.0%	6; 9.7%	8; 12.3%	0.64
Chronic alcohol use	1; 0.79%	0; 0%	1; 1.5%	0.33
Chronic tobacco use	6; 4.7%	1; 1.6%	5; 7.7%	0.11
Obesity	32; 25.2%	11; 17.7%	21; 32.3%	0.06
COVID-19 vaccination status (*n*, %):				
Full immunisation (≥ 3 doses)	87; 68.5%	34; 54.8%	53; 81.5%	< 0.01
Partial immunisation (< 3 doses)	30; 23.6%	21; 33.9%	9; 13.8%	0.01
No data (*n*, %)	10; 7.9%	7; 11.3%	3; 4.6%	0.16
Previously documented SARS-CoV-2 infection (*n*, %):				
Within 3 months	2; 1.6%	2; 3.2%	0; 0%	0.14
Over 3 months	8; 6.3%	6; 9.7%	2; 3.1%	0.13
No previous infection	49; 38.6%	24; 38.7%	25; 38.5%	0.98
No data	68; 53.5%	30; 48.4%	38; 58.5%	0.26
COVID-19 severity (*n*, %):				
Asymptomatic	44; 34.6%	19; 30.6%	25; 38.5%	0.35
Non-severe	82; 64.6%	42; 67.7%	40; 64.5%	0.47
Severe	1; 0.8%	1; 1.6%	0; 0%	0.30
SARS-CoV-2 RNAemia positivity* (*n*, %)	30/117; 25.6%	16/56; 28.6%	14/61; 23.0%	0.43
Lung infiltration typical for COVID-19 on chest CT scan* (*n*, %)	18/102; 17.6%	9/55; 16.4%	9/47; 19.1%	0.52
Time from symptom onset to remdesivir (days, median; IQR; min–max)	3; 3; 0–63	4; 5; 0–63	3; 2; 0–14	0.13
Time from first positive respiratory SARS-CoV-2 PCR to remdesivir (days, median; IQR; min–max)	0; 1; 0–26	0; 1; 0–26	0; 1; 0–4	0.35
Time from first positive to first negative respiratory SARS-CoV-2 PCR (days, median; IQR; min–max)	22; 19; 0–78	27; 16; 5–78	15; 8; 0–45	0.20
Clinical outcomes (*n*, %)				
Need for hospitalisation due to COVID-19	9; 7.1%	9; 14.5%	0; 0%	< 0.01
Need for oxygen supplementation	3; 2.4%	3; 4.8%	0; 0%	0.07
Need for intensive care unit admission	2; 1.6%	2; 3.2%	0; 0%	0.14
All-cause death	1; 0.79%	1; 1.6%	0; 0%	0.30
Administered doses of remdesivir (*n*, %)				
Standard course (3 days)	49; 38.6%	8; 12.9%	41; 63.1%	< 0.01
Prolonged course (5 days)	78; 61.4%	54; 87.1%	24; 36.9%	< 0.01

n.a., not applicable

^*^ In proportion to the number of tested patients

## Discussion

### Present study

This single-centre prospective cohort study evaluated clinical characteristics and outcomes of high-risk adult outpatients receiving early remdesivir therapy in the SARS-CoV-2 omicron variant era. We documented low rates of hospitalisation and all-cause death, and all negative outcomes occurring among patients with malignant haematological diseases.

### Current literature

In the literature, multiple studies have assessed the therapeutic effect of an early 3-day remdesivir therapy among adult outpatients with a wide-spectrum of patient-level risk factors (Gottlieb et al. 2022; Panagopoulos et al. 2022). A randomised, double-blind, placebo-controlled study involving 562 nonhospitalised high-risk patients documented an 87% survival among patients receiving the 3-day remdesivir therapy compared to the placebo arm (Gottlieb et al. 2022). Also, a matched-pair retrospective study comparing COVID-19 related hospitalisation and acute respiratory failure among outpatients treated with a 3-day remdesivir to placebo reported a 75% lower rate of hospital admission and a 95% lower rate of acute respiratory failure among patients receiving remdesivir (Panagopoulos et al. 2022).

Clinical data concerning SARS-COV-2-infected patients with haematological malignancies during the omicron wave reports a higher risk for progression to severe disease and a reduced immune response post-vaccination, but decreased hospital admission and mortality rates were also documented (Blennow et al. 2022). Different comprehensive strategies such as remdesivir plus monoclonal antibodies or remdesivir plus convalescent plasma therapy proved to be successful for protection among patients with an active haematological malignancy (Cesaro et al. 2022). However, efficacy of monoclonal antibodies depends on the circulating variant, whereas accessibility of convalescent plasma therapy may be hindered in some low-income settings (IDSA 2022).

During the alpha and delta waves, monoclonal antibody therapies were generally available for the treatment of SARS-CoV-2-infected high-risk outpatients (IDSA 2022). In addition, in Hungary, favipiravir had been included in the national COVID-19 guideline based on an off-label approval by the National Institute of Pharmacy and Nutrition. Subsequent trials, however, did not confirm its positive effect on clinical outcomes in the early phase of COVID-19 (Szabo 2021; Chuah et al. 2022).

So far, two orally administered antivirals became available in some European countries with full or conditional approval by the European Medicine Agency*.* The cysteine protease inhibitor nirmatrelvir/ritonavir and the replication-inhibiting molnupiravir proved to be effective not only in in vitro studies but also in real-life settings during the PANGO BA.2 dominance (IDSA 2022; WHO 2022). These drugs are easy to administer and possess good bioavailability with generally acceptable side-effect profiles (IDSA 2022; WHO 2022). However, in countries with limited accessibility to oral antivirals against SARS-CoV-2, administration of remdesivir may be retained as an essential treatment strategy for COVID-19. Furthermore, among patients with haematological malignancies where viral persistence, longer viral shedding, and viraemia are a problematic tendency, a parenterally administered medication could be a rational option (Cesaro et al. 2022). This might be mirrored by the change in WHO recommendations where indication for remdesivir was expanded to the outpatient setting while also acknowledging its efficacy against SARS-CoV-2 PANGO variants BA.2, BA.4, and BA.5 (Gottlieb et al. 2022; WHO 2022).

### Study limitations

The present study has some limitations. First, we did not identify SARS-CoV-2 variants of each patient. We considered the omicron variant of SARS-CoV-2 as dominant in Hungary based on surveillance samples to the European Centre for Disease Control and Prevention by national authorities (ECDC 2022). Second, a relatively small number of patients were involved in the study. In addition, as the study design is a simple observational study, no randomisation or placebo control was feasible in the outpatient setting for high-risk patients. In a pandemic situation with a high influx of patients per day, a throughout patient medical history taking was not always feasible; therefore, we might assume that some clinical data were biased during collection. The majority of patients in the cohort were at least partially vaccinated, which may partially account for lower hospitalisation and mortality rates. Lastly, we did not follow severe or critical cases from the study population over the pre-defined 28-day period; therefore, late outcomes could not be reported in our study.

## Conclusion

In this single-centre prospective cohort study conducted during the SARS-CoV-2 omicron wave in the outpatient setting, outcomes of high-risk adults, including patients with haematological malignancies, treated with early remdesivir therapy were favourable, providing a clinically feasible strategy against COVID-19.

## Supplementary Information

Below is the link to the electronic supplementary material.

Supplementary file1 (DOCX 18 KB)

## Data Availability

Anonymised data of patients are available from the corresponding author upon reasonable request.

## Abbreviations

COVID-19: Coronavirus disease 2019
CT: Computed tomography
IQR: Interquartile range
PANGO: Phylogenetic assignment of named global outbreak
PCR: Polymerase chain reaction
QD: Once daily [*quaque** die*]
RT-PCR: Real-time polymerase chain reaction
SARS-CoV-2: Severe acute respiratory syndrome coronavirus 2
WHO: World Health Organisation
