# Supplementary material for: Outcomes of high-risk adult outpatients treated with early remdesivir therapy during the severe acute respiratory syndrome coronavirus 2 (SARS-CoV-2) omicron era: experiences from the national centre of Hungary
Source: Naunyn Schmiedebergs Arch Pharmacol. 2023 Mar 9;396(8):1857–62. doi: 10.1007/s00210-023-02456-y (PMC9995722; doi:10.1007/s00210-023-02456-y)
Supplement: Supplementary file 1 — Supplementary file1 (DOCX 18 KB) [file 210_2023_2456_MOESM1_ESM.docx]

**Supplementary material 1. Concise standard operating procedure at the COVID-19 Outpatient Department of South Pest Central Hospital, National Institute of Haematology and Infectious Diseases (Budapest, Hungary)**

The *Corona Virus Disease 2019* (COVID-19) Outpatient Department is a national-level referral institution operating with 5 working physicians continually each day. The department is located at South Pest Central Hospital, National Institute of Haematology and Infectious Diseases (Albert Flórián Street 5-7., H-1097, Budapest, Hungary).

Patients treated at our COVID-19 Department are referred by their general practitioner or physician of any subspecialty. After admission, patients are examined physically, and their past medical history is collected. Then, standard laboratory tests, respiratory *Severe Acute Respiratory Syndrome Coronavirus 2* (SARS-CoV-2) real-time polymerase chain reaction (RT-PCR) tests and chest CT scans are performed. COVID-19 infection severity is assessed in accordance with the *World Health Organization* (WHO) criteria [1].

Asymptomatic or symptomatic patients with a positive respiratory SARS-CoV-2 RT-PCR and with ≥1 pre-defined risk factors for disease progression, but without hospitalisation requirement, are offered the 3-day remdesivir intravenous outpatient therapy. Risk factors for COVID-19 progression are essential hypertension, obesity (body mass index >25 kg/m2), chronic cardiovascular disease, chronic cerebrovascular disease, chronic pulmonary disease, chronic renal disease, chronic liver disease, diabetes mellitus, immunocompromised states, and active onco-haematological malignancy [1]. After informed consent is obtained, therapy is promptly initiated *per protocol*.

During the 3-day therapy, attending physicians re-evaluate the clinical state and symptoms of patients upon daily *in-person* visits. After end of treatment (EOT), patients are requested to report for an unscheduled *in-person* visit, or by telephone or e-mail, in case their clinical state deteriorated or a new symptom emerged. At EOT+28 days, follow-up is completed by assessing patient status in the National eHealth Infrastructure (social security database of Hungary).

**References**

[1] World Health Organisation. Living guidance for clinical management of COVID-19. https://apps.who.int/iris/bitstream/handle/10665/349321/WHO-2019-nCoV-clinical-2021.2-eng.pdf. Accessed 22. October 2022.
